# Hypernatremia during the first week of life in very preterm infants and neurodevelopmental outcomes at 3 to 4 years of age: a cohort study

**DOI:** 10.1186/s12887-026-06571-6

**Published:** 2026-02-03

**Authors:** Michiko Murakami, Kei Tamai, Naomi Matsumoto, Akihito Takeuchi, Makoto Nakamura, Takashi Yorifuji, Misao Kageyama

**Affiliations:** 1 Division of Neonatology, NHO Okayama Medical Center, 1711-1 Tamasu, Kita-ku, Okayama, 701-1192 Japan; 2 Department of Epidemiology, Faculty of Medicine, Dentistry and Pharmaceutical Sciences, Okayama University, Okayama, Japan

**Keywords:** Hypernatremia, Development, Very preterm, Cerebral palsy

## Abstract

**Background:**

Hypernatremia is a common electrolyte disorder in both term and preterm infants. Previous studies have suggested a correlation between hypernatremia and short-term complications in preterm infants, such as intraventricular hemorrhage and chronic lung disease. However, the relationship between hypernatremia and neurodevelopmental outcomes is less well understood. This study aimed to assess the association between hypernatremia during the first week of life and neurodevelopmental outcomes at 3–4 years of age in very preterm infants.

**Methods:**

This single-center, retrospective cohort study analyzed data from preterm infants born at less than 32 weeks of gestation between 2010 and 2020. Infants with peak whole blood sodium levels > 145 mEq/L during the first week of life were included in the hypernatremia group and those with ≤ 145 mEq/L in the non-hypernatremia group. The primary outcome was neurodevelopmental impairment (NDI) at 3–4 years of age, defined as developmental impairment (developmental quotient < 70), cerebral palsy, hearing impairment, or visual impairment. Secondary outcomes were the components of the primary outcome. We conducted Poisson regression analyses with robust variance, adjusting for perinatal confounders.

**Results:**

Of 272 infants with neurodevelopmental data, 82 and 190 infants were in the hypernatremia and non-hypernatremia groups, respectively. The median (interquartile range) gestational age and birth weight were 26.4 (25.1–28.0) and 28.7 (26.6–30.3) weeks and 860 (670–1062) and 997 (778–1264) g for infants in the hypernatremia and non-hypernatremia groups, respectively. Infants in the hypernatremia group had a greater incidence of NDI (29.3% vs. 14.7%, adjusted risk ratio [RR] 1.75, 95% CI 1.08–2.84) and cerebral palsy (8.5% vs. 1.6%, adjusted RR 5.5, 95% CI 1.72–17.63) than those in the non-hypernatremia group.

**Conclusions:**

Hypernatremia during the first week of life was associated with an increased risk of NDI at 3–4 years of age in very preterm infants.

**Supplementary Information:**

The online version contains supplementary material available at 10.1186/s12887-026-06571-6.

## Background

Hypernatremia, defined as a serum sodium level > 145 mEq/L [1], is a common electrolyte disorder in both term and preterm infants. Preterm infants tend to have hypernatremia because of their large body surface area-to-body weight ratio and immature skin, which causes high insensible water loss; low urine concentrating capacity due to immature renal function is a factor as well [2]. The incidence of hypernatremia during the first 3 days of life is as high as 40% in preterm infants born at 29 weeks of gestation [3].

Hypernatremia has been linked to poorer neurological outcomes in both adult and pediatric intensive care patients [4–6]. In preterm infants, it is associated with intraventricular hemorrhage, chronic lung disease, patent ductus arteriosus, and necrotizing enterocolitis [7–10]. A retrospective observational study in the United States demonstrated an association between hypernatremia in the first week of life and lower fine motor ability at 18 months of corrected age in infants born at less than 32 weeks of gestational age [11]. The association between hypernatremia and outcomes beyond 18 months of age in preterm infants has not been previously examined, to the best of our knowledge. This study aimed to examine the association between hypernatremia during the first week of life and neurodevelopmental outcomes at 3 to 4 years of age in preterm infants born at less than 32 weeks of gestation.

## Materials and methods

### Study cohort

We retrospectively examined very preterm infants (born at less than 32 weeks of gestational age) who were admitted to our tertiary neonatal intensive care unit (NICU) between January 2010 and December 2020. Those who were born outside of our hospital, had major congenital anomalies, or died within 7 days of age were excluded. The study was approved by the institutional review board of our institution under protocol number RINKEN 2024-005. Owing to the retrospective nature of the study and the use of anonymized data, the requirement for written informed consent from parents was waived. An opt-out provision was provided.

### Data collected

Clinical data were collected from discharge summaries and cross-verified using admission charts. Intraventricular hemorrhage was detected using daily cranial ultrasonography and confirmed via cranial magnetic resonance imaging (MRI) before discharge. Severe intraventricular hemorrhage was categorized as grade 3 or higher based on the criteria established by Papile et al. [12]. An abnormal MRI finding of the brain was defined as the presence of any of the following findings on the pre-discharge MRI: white matter injury, delayed myelination, or cerebellar hemorrhage. Periventricular leukomalacia was diagnosed using cranial ultrasonography or MRI. Bronchopulmonary dysplasia was defined as the need for supplemental oxygen or positive pressure ventilation at a postmenstrual age of 36 weeks. Necrotizing enterocolitis was defined as necrotizing enterocolitis Bell’s stage 2 or higher [13]. Retinopathy of prematurity was assessed and managed using the Japanese Ministry of Health, Labour, and Welfare Task Force guidelines.

Gestational age was determined using ultrasonography during the first trimester and the date of the last menstrual period. Small for gestational age was defined as birth weight below the tenth percentile for gestational age using standard Japanese neonatal anthropometric charts [14]. Antenatal steroid use was defined as the administration of any dose of corticosteroids to the mother before delivery. Use of postnatal corticosteroids and non-steroidal anti-inflammatory drugs was defined as the administration of any dose before measurement of the peak sodium level within the first week of life.

### NICU policy for fluid and electrolyte administration and incubator settings

All infants received standardized fluid treatments according to our NICU protocol: 60 mL/kg on day 1, 70–80 mL/kg on day 2, 80–100 mL/kg on day 3, and 100–120 mL/kg from day 4 onward. If the whole blood sodium level exceeded 150 mEq/L, the fluid volume was increased by 10–20 mL/kg/day. Intravenous sodium supplementation was initiated between 3 and 5 days of age and gradually increased to approximately 4 mEq/kg/day. The initial incubator settings are detailed in Table 1. The bedside nurse subsequently adjusted the incubator settings based on the infant’s age, skin condition, and body temperature.

**Table 1:** NICU policy for initial incubator settings in very preterm infants

Birth weight, g	Gestational age, weeks	Temperature in incubator, ℃	Humidity in incubator, %
< 850	< 26	37.0	95
850–999	26	37.0	90
1000–1249	27–29	35.5–35.6	80
1250–1500	30–31	34.0–35.0	75

### Whole blood sodium measurements

In our NICU, whole blood sodium levels of very preterm infants are measured daily during the first week of life. These levels from birth through 7 days of life were collected retrospectively from the admission records. Infants were classified according to the highest sodium level measured during the first week of life into hypernatremia (> 145 mEq/L) and non-hypernatremia (≤ 145 mEq/L) groups. Levels were measured using the ABL700 blood gas analyzer (Radiometer, Copenhagen, Denmark) from 2010 to March 20, 2015, and the ABL90 analyzer (Radiometer) from March 21, 2015 to 2020. We did not differentiate between blood sample sources (arterial, venous, or capillary).

### Neurodevelopmental outcomes

A comprehensive neurodevelopmental assessment was conducted at age 3 to 4 years. To assess developmental progress, certified psychologists administered the Kyoto Scale of Psychological Development 2001. Developmental impairment was defined as an overall developmental quotient of less than 70, which is reportedly equivalent to a Bayley III cognitive score of less than 85 [15]. Cerebral palsy was defined as the presence of abnormal muscle tone in one or more extremities accompanied by atypical control of movement and posture [16]. Visual impairment was defined as blindness in one or both eyes. Hearing impairment was defined as the need for hearing aids. Neurodevelopmental impairment (NDI) was defined as the presence of developmental impairment, cerebral palsy, or visual or hearing impairment. The primary outcome was NDI. Secondary outcomes were the individual components of the primary outcome.

### Statistical analysis

Patient characteristics were compared between the hypernatremia and non-hypernatremia groups. To assess the impact of loss to follow-up, characteristics were also compared between those with and without neurodevelopmental data at age 3 to 4 years. The differences in the proportion between the two groups were tested using the χ2 test or the Mann-Whitney U test. Robust Poisson regression was performed to investigate the associations between hypernatremia and the primary and secondary outcomes. After adjusting for potential confounders, the non-hypernatremia group was used as the reference to estimate risk ratios (RRs) with 95% confidence intervals (CIs). Potential confounders were selected based on previous studies [11, 17] and included gestational age (categorized as 22–24, 25–27, and 28–31 weeks), sex (binary), small for gestational age (binary), antenatal corticosteroids (binary), maternal age (categorized as ≤ 24, 25–29, 30–34, and ≥ 35 years), postnatal corticosteroids use (binary), and non-steroidal anti-inflammatory drugs use (binary).

Restricted cubic spline analysis was performed to evaluate the relationship between the highest sodium value during the first week of life and neurodevelopmental outcomes at 3 to 4 years of age. We inserted knots at 140, 145, and 150 mEq/L and controlled for the same covariates as in the original categorical analyses. Adjusted RRs with 95% CIs were obtained for each sodium value using 140 mEq/L as a reference from the spline analyses.

In the sensitivity analysis, the association between hypernatremia and neurodevelopmental outcome was examined with patients stratified according to gestational age: 22–27 weeks and 28–31 weeks. We also tested for an interaction between hypernatremia and gestational age (22–27 vs. 28–31 weeks) using a Poisson regression model. The model was further adjusted for mode of delivery (cesarean or vaginal) and Apgar score at 5 min (< 7 or ≥ 7), along with the covariates from the primary analysis. These factors were theoretically considered potential confounders that had not been accounted for in previous studies. Moreover, we performed additional analyses, including stratified analyses by the number of days with hypernatremia (1 day and ≥ 2 days) and by the change in the blood sodium analyzer (ABL700, 2010–March 20, 2015; ABL90, March 21, 2015–2020), as well as a sensitivity analysis excluding infants with severe hyponatremia (< 125 mEq/L) [18, 19] during the first week of life.

Statistical analyses were performed using Stata SE version 18 (Stata Corp LLC, College Station, TX, USA). P values less than 0.05 were considered significant.

## Results

**Fig. 1:**
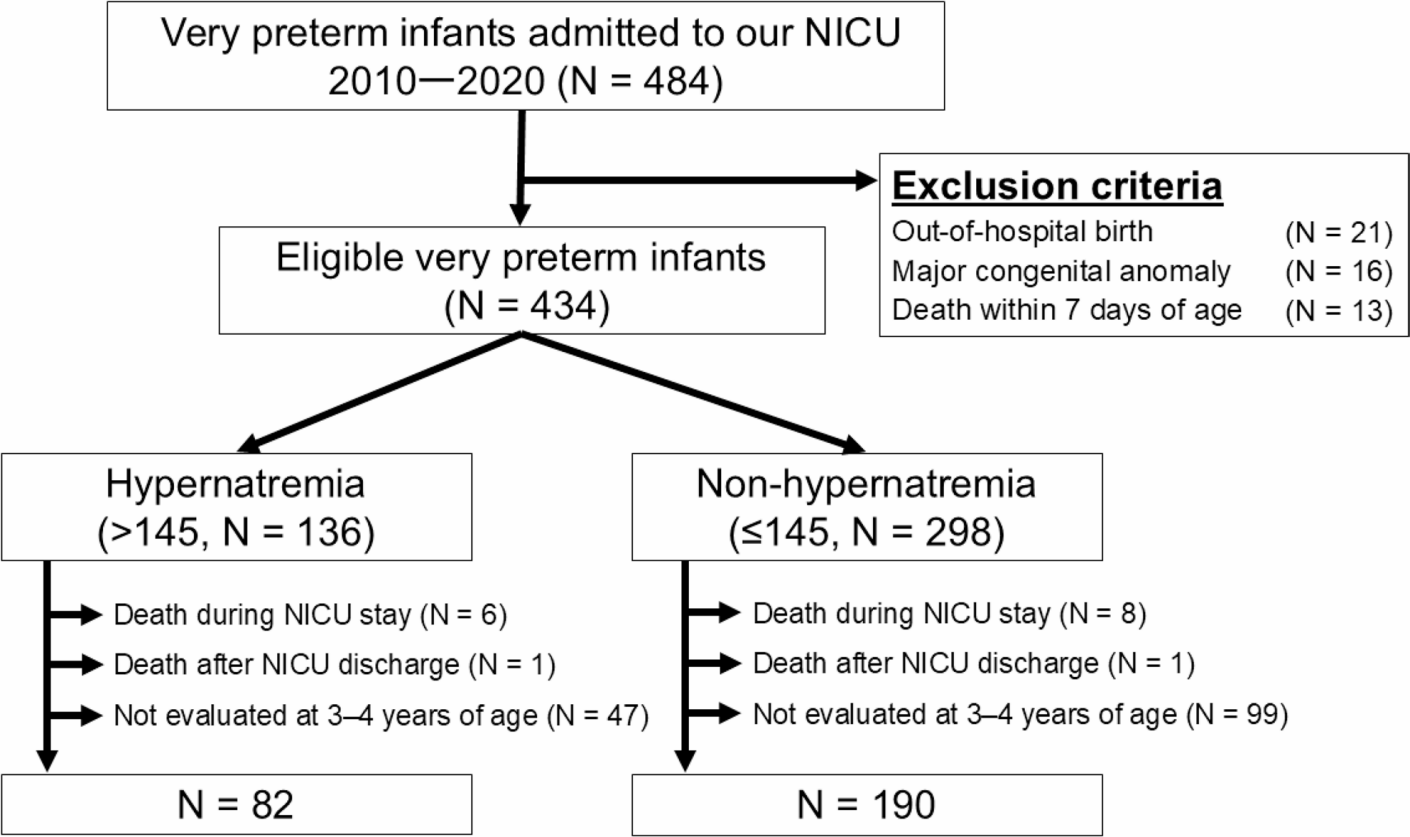
Study flowchart. Abbreviations: NICU, neonatal intensive care unit

Between 2010 and 2020, 484 very preterm infants were admitted to the NICU. Fifty were excluded owing to out-of-hospital birth (*n* = 21), major congenital anomalies (*n* = 16), and death within 7 days of age (*n* = 13). Among the remaining 434 infants, the peak sodium level during the first week of life was > 145 and ≤ 145 mEq/L in 136 (31%) and 298 (69%) patients, respectively. Fourteen of the 434 died before NICU discharge and two died after NICU discharge; these cases were excluded from the analysis as neurodevelopmental testing could not be performed. Neurodevelopmental data at age 3 to 4 years were not available in 146 infants. Finally, among those with available neurodevelopmental data, 82 infants in the hypernatremia group and 190 in the non-hypernatremia group were analyzed. A study flow chart is shown in Fig. 1.

Characteristics of patients with available neurodevelopmental data according to group are shown in Table 2. Gestational age, birth weight, and the prevalence of an Apgar score < 7 at 5 min were lower in the hypernatremia group. In contrast, the prevalence rates of postnatal corticosteroid use, vaginal delivery, and retinopathy of prematurity requiring treatment were higher in the hypernatremia group.

**Table 2:** Characteristics of patients with available neurodevelopmental data according to group

	Hypernatremia group	Non-hypernatremia group	*P* value^a^
	*N* = 82	*N* = 190	
Gestational age, weeks	26.4 (25.1–28.0)	28.7 (26.6–30.3)	< 0.01
Birth weight, g	860 (670–1062)	997 (778–1264)	< 0.01
Small for gestational age	14 (17.1)	47 (24.7)	0.16
Sex, male	47 (57.3)	96 (50.5)	0.30
Antenatal corticosteroid use	76 (92.7)	162 (85.3)	0.09
Postnatal corticosteroid use	9 (11.0)	4 (2.1)	< 0.01
Non-steroidal anti-inflammatory drug use	6 (7.3)	16 (8.4)	0.76
Maternal age, years			0.30
< 25	9 (11.0)	14 (7.4)	
25–29	17 (20.7)	44 (23.2)	
30–34	25 (30.5)	76 (40.0)	
≥ 35	31 (37.8)	56 (29.5)	
Mode of delivery			< 0.01
Cesarean delivery	41 (50.0)	128 (67.4)	
Vaginal delivery	41 (50.0)	62 (32.6)	
Apgar score at 5 min, <7	44 (53.7)	129 (67.9)	0.03
Severe intraventricular hemorrhage (grade 3 and 4)	3 (3.7)	4 (2.1)	0.46
Intraventricular hemorrhage (all grades)	21 (25.6)	31 (16.3)	0.07
Periventricular leukomalacia	2 (2.4)	2 (1.1)	0.38
Bronchopulmonary dysplasia	17 (20.7)	32 (16.8)	0.44
Necrotizing enterocolitis	4 (4.9)	5 (2.6)	0.34
Retinopathy of prematurity requiring treatment	24 (29.3)	31 (16.3)	0.02
Abnormal brain MRI findings^b^	1/76 (1.3)	5/162 (3.1)	0.42

Data are expressed as numbers (%) or medians (interquartile range)

^a^The differences between the hypernatremia and non-hypernatremia groups were tested using the χ2 test or Mann-Whitney U test

^b^Defined as any of white matter injury, delayed myelination, or cerebellar hemorrhage on pre-discharge MRI

Patients without neurodevelopmental data at age 3 to 4 years had higher gestational age and birth weight, younger mothers, and a lower prevalence of an Apgar score < 7 at 5 min than those with neurodevelopmental data (Table 3).

**Table 3:** Patient characteristics according to neurodevelopmental data availability

	With neurodevelopmental data at 3–4 years of age	Without neurodevelopmental data at 3–4 years of age	*P* value^a^
	*N* = 272	*N* = 146	
Gestational age, weeks	28.0 (26.0–29.8)	29.8 (27.3–31.0)	< 0.01
Birth weight, g	946 (746–1216)	1238 (844–1490)	< 0.01
Small for gestational age	61 (22.4)	24 (16.4)	0.13
Sex, male	143 (52.6)	86 (58.9)	0.22
Antenatal corticosteroid use	238 (87.5)	121 (82.9)	0.20
Postnatal corticosteroid use	13 (4.8)	7 (4.8)	0.99
Non-steroidal anti-inflammatory drug use	22 (8.1)	5 (3.4)	0.06
Maternal age, years			0.02
< 25	23 (8.5)	23 (15.8)	
25–29	61 (22.4)	43 (29.5)	
30–34	101 (37.1)	43 (29.5)	
≥ 35	87 (32.0)	37 (25.3)	
Mode of delivery			0.29
Cesarean delivery	169 (62.1)	83 (56.8)	
Vaginal delivery	103 (37.9)	63 (43.2)	
Apgar score at 5 min, <7	99 (36.4)	37 (25.3)	0.02
Hypernatremia group	82 (30.1)	47 (32.2)	0.67

Data are expressed as numbers (%) or medians (interquartile range)

^a^The differences between the groups with and without data of neurodevelopmental data at age 3–4 years were tested using the χ2 test or Mann-Whitney U test

Table 4 shows the incidence and risk of the primary and secondary outcomes at age 3 to 4 years in the hypernatremia and non-hypernatremia groups. The incidence rates of NDI (29.3% vs. 14.7%) and cerebral palsy (8.5% vs. 1.6%) were higher in the hypernatremia group. After adjusting for potential confounders, the risks of both NDI (adjusted RR, 1.75; 95% CI, 1.08–2.84) and cerebral palsy (adjusted RR, 5.5; 95% CI, 1.72–17.63) were higher in the hypernatremia group.

**Table 4:** Incidence and risk of the primary and secondary outcomes in the hypernatremia versus non-hypernatremia groups

	*N* case/*N* total	(%)	Crude RR	(95% CI)	Adjusted RR	(95% CI)^a^
Neurodevelopmental impairment^b^						
Non-hypernatremia group	28/190	(14.7)	1	(reference)	1	(reference)
Hypernatremia group	24/82	(29.3)	1.99	(1.23–3.21)	1.75	(1.08–2.84)
Developmental impairment^c^						
Non-hypernatremia group	27/190	(14.2)	1	(reference)	1	(reference)
Hypernatremia group	21/82	(25.6)	1.80	(1.08–3.00)	1.56	(0.95–2.58)
Cerebral palsy						
Non-hypernatremia group	3/190	(1.6)	1	(reference)	1	(reference)
Hypernatremia group	7/82	(8.5)	5.41	(1.43–20.44)	5.50	(1.72–17.63)
Visual or hearing impairment						
Non- hypernatremia group	0/190	(0)	1	(reference)	1	(reference)
Hypernatremia group	1/82	(1.2)	NE		NE	

*Abbreviations:* *CI* Confidence interval, *NE* Not estimable, *RR* Risk ratio

^a^Adjusted for gestational age, sex, small for gestational age, antenatal corticosteroids, maternal age, postnatal corticosteroids use, and non-steroidal anti-inflammatory drugs use

^b^Infants with developmental impairment, cerebral palsy, or visual or hearing impairment

^c^Infants with an overall Developmental Quotient score < 70 on the Kyoto Scale of Psychological Development

**Fig. 2:**
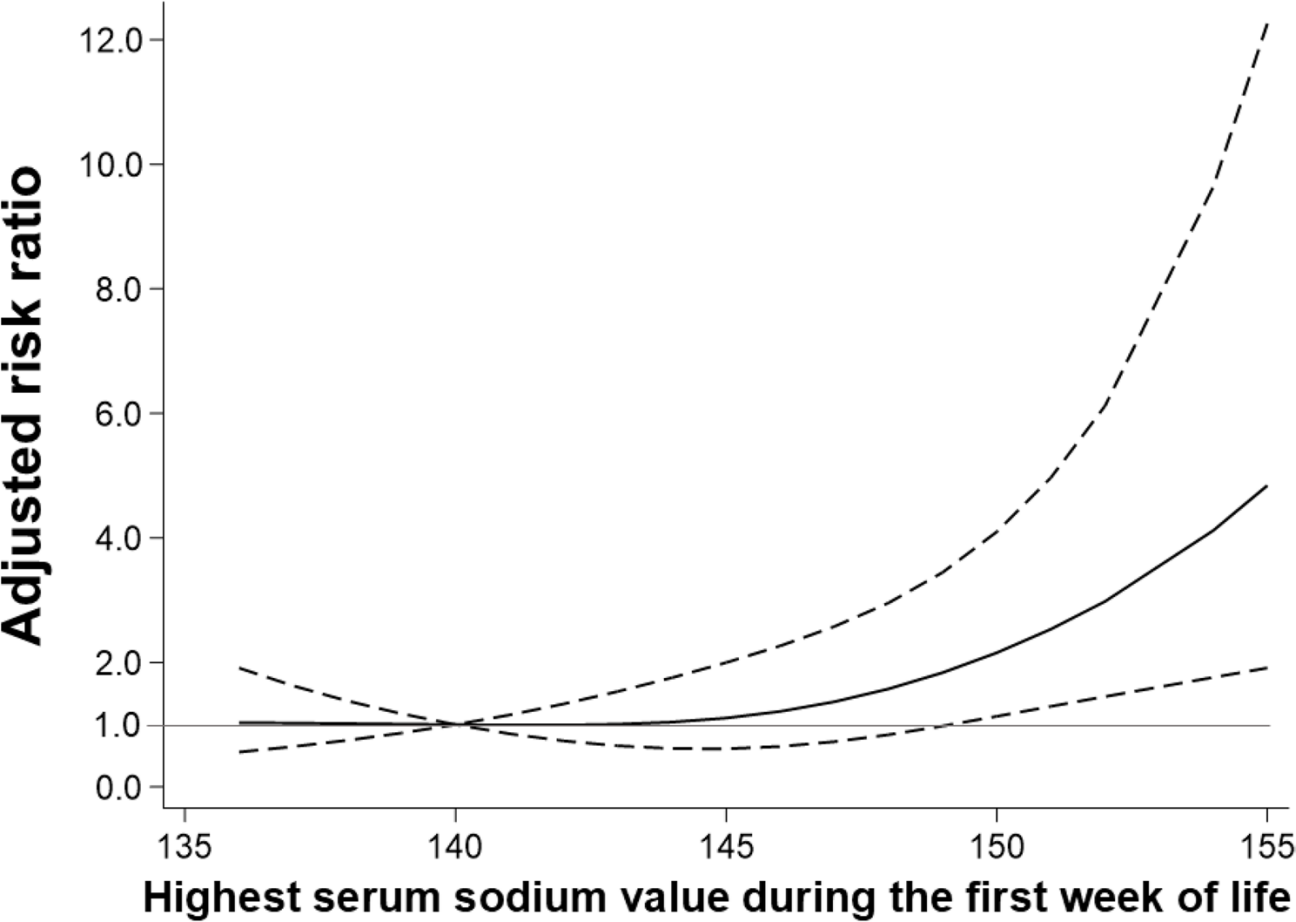
Restricted cubic spline analysis. Adjusted risk ratios (solid line) and 95% confidence intervals (dotted lines) for the association between the highest sodium value in the first week of life and neurodevelopmental impairment. Highest sodium levels above 150 mEq/L were associated with an increased risk of neurodevelopmental impairment

The results from the cubic spline analysis are shown in Fig. 2. Highest sodium levels in the first week of life above 150 mEq/L were associated with an increased risk of NDI

When patients were stratified by gestational age, the findings in infants born at 22–27 weeks of gestation were similar to those reported above (Table 5). However, among those born at 28–31 weeks, the adjusted risks of NDI for hypernatremia disappeared. The interaction between hypernatremia and gestational age was not statistically significant for the primary outcome (NDI) (*p* = 0.49), suggesting no evidence that the association differs by gestational age group.

**Table 5:** Associations between hypernatremia status and primary and secondary neurodevelopmental outcomes, stratified by gestational age

	22–27 weeks of gestational age				28–31 weeks of gestational age			
	Hypernatremia group		Non-hypernatremia group		Hypernatremia group		Non-hypernatremia group	
	N = 59		N = 75		N = 23		N = 115	
Neurodevelopmental impairment^b^								
N case/N total (%)	19/59	(32.2)	12/75	(16.0)	5/23	(21.7)	16/115	(13.9)
Adjusted RR (95% CI)^a^	2.04	(1.06–3.94)	1	(reference)	0.96	(0.30–3.07)	1	(reference)
Developmental impairment^c^								
N case/N total (%)	17/59	(28.8)	12/75	(16.0)	4/23	(17.4)	15/115	(13.0)
Adjusted RR (95% CI)^a^	1.83	(0.92–3.61)	1	(reference)	0.61	(0.19–1.97)	1	(reference)
Cerebral palsy								
N case/N total (%)	6/59	(10.2)	1/75	(1.3)	1/23	(4.3)	2/115	(1.7)
Adjusted RR (95% CI)^a^	8.01	(1.76–36.37)	1	(reference)	2.63	(0.36–19.40)	1	(reference)
Visual or hearing impairment								
N case/N total (%)	1/59	(1.7)	0/75	(0)	0/23	(0)	0/115	(0)
Adjusted RR (95% CI)^a^	NE		1	(reference)	NE		1	(reference)

*Abbreviations:* *CI* Confidence interval, *NE* Not estimable, *RR* Risk ratio

^a^Adjusted for gestational age, sex, small for gestational age, antenatal corticosteroids, maternal age, postnatal corticosteroids use, and non-steroidal anti-inflammatory drugs use

^b^Infants with developmental impairment, cerebral palsy, or visual or hearing impairment

^c^Infants with an overall Developmental Quotient score <70 on the Kyoto Scale of Psychological Development

Including mode of delivery and 5-minute Apgar score as potential confounders in the sensitivity analysis did not substantially alter the main findings (Table 6). When the hypernatremia group was divided by the number of days with hypernatremia during the first week of life (1 day vs. ≥ 2 days, not necessarily consecutive), the increased risks of NDI, developmental impairment, and cerebral palsy were observed only among infants with hypernatremia on ≥ 2 days, but not among those with hypernatremia on a single day (Supplementary Table 1). Supplementary Tables 2 and 3 present additional analyses stratified by the type of blood gas analyzer used (ABL700 and ABL90) and excluding infants with severe hyponatremia (< 125 mEq/L; *n* = 14). In these analyses, infants with hypernatremia tended to have higher point estimates of RR for NDI, although the CIs were wide and overlapped.

**Table 6:** Associations between hypernatremia and neurodevelopmental outcomes adjusted for mode of delivery and 5-minute Apgar score

	*N* case/*N* total	(%)	Crude RR	(95% CI)	Adjusted RR	(95% CI)^a^
Neurodevelopmental impairment^b^						
Non-hypernatremia group	28/190	(14.7)	1	(reference)	1	(reference)
Hypernatremia group	24/82	(29.3)	1.99	(1.23–3.21)	1.69	(1.04–2.77)
Developmental impairment^c^						
Non-hypernatremia group	27/190	(14.2)	1	(reference)	1	(reference)
Hypernatremia group	21/82	(25.6)	1.80	(1.08–3.00)	1.50	(0.89–2.51)
Cerebral palsy						
Non-hypernatremia group	3/190	(1.6)	1	(reference)	1	(reference)
Hypernatremia group	7/82	(8.5)	5.41	(1.43–20.44)	6.21	(1.70–22.66)
Visual or hearing impairment						
Non-hypernatremia group	0/190	(0)	1	(reference)	1	(reference)
Hypernatremia group	1/82	(1.2)	NE		NE	

*Abbreviations:* *CI* Confidence interval, *NE* Not estimable, *RR* Risk ratio

^a^Adjusted for gestational age, sex, small for gestational age, antenatal corticosteroid use, maternal age, postnatal corticosteroid use, non-steroidal anti-inflammatory drug use, mode of delivery, and 5-minute Apgar score.

^b^Infants with developmental impairment, cerebral palsy, or visual or hearing impairment

^c^Infants with an overall Developmental Quotient score < 70 on the Kyoto Scale of Psychological Development

## Discussion

In this study, we investigated the association between hypernatremia during the first week of life and neurodevelopmental outcomes at 3 to 4 years of age among very preterm infants. Infants in the hypernatremia group had a greater risk of NDI and cerebral palsy than those in the non-hypernatremia group. The spline analysis results indicated that the risk of NDI increased as the sodium level increased over 150 mEq/L.

Several studies have reported that hypernatremia in critically ill adults and children is associated with an increased risk of mortality and worse neurological outcomes [6, 20, 21]. However, the relationship between hypernatremia in a neonate’s first week of life and neurodevelopmental outcomes is less well understood. A case-control study from Iran reported that the incidence of developmental delay at 2 years of age was higher in term infants with hypernatremia (serum sodium concentration ≥ 150 mEq/L) than those with serum sodium concentration < 150 mEq/L (12% vs. 0%) [22]. Similarly, a retrospective cohort study of very preterm infants in the United States reported that Bayley III fine motor neurodevelopmental scores at 18 months of corrected age were significantly lower in the hypernatremia group (serum sodium > 145 mEq/L) than the non-hypernatremia group (8.97 ± 2.13 vs. 10.08 ± 1.98) [11]. A Canadian retrospective cohort study also suggested that greater fluctuations in glucose-corrected plasma sodium levels during the first days of life in very preterm infants were associated with an increased risk of death or neurodevelopmental impairment at 18 months of corrected age, even after adjusting for gestational age (B = 2.1; 95% CI, 0.16–4.04) [17]. The impact of hypernatremia in very preterm infants on neurodevelopmental outcomes beyond 1.5 years of age has not been explored in previous studies. In our study, very preterm infants with hypernatremia (> 145 mEq/L) during the first week of life had a higher risk of NDI at 3 to 4 years of age than those without hypernatremia.

The association between hypernatremia in the first week of life and neurodevelopmental outcomes in very preterm infants could be due to a rapid loss of water from brain cells during periods of hypernatremia or an abrupt decrease in extracellular fluid osmolality during reversal of hypernatremia, both of which may contribute to brain edema, although this remains a speculative explanation. An increase in extracellular fluid sodium concentration raises its tonicity, creating a hypertonic state. This drives an osmotic shift of water from the intracellular to the extracellular space via the osmotic gradient, reducing intracellular volume and causing cell shrinkage [23]. Cellular adaptations in the brain to restore fluid balance through osmolyte production take approximately a week, preserving cell volume and stabilizing proteins [24]. However, in acute hypernatremia, the rapid loss of water from brain cells might distort their structure, potentially leading to complications such as intracerebral hemorrhage [25], venous sinus thrombosis, and infarction [26]—conditions that could result in death or permanent neurological sequelae. In our cohort, intraventricular hemorrhage was observed, although no cases of brain edema, venous thrombosis, or infarction were identified. Several studies have reported an association between hypernatremia and intraventricular hemorrhage in preterm infants, suggesting a possible pathway through which hypernatremia contributes to adverse neurodevelopmental outcomes [9, 27]. Furthermore, a rapid reduction in extracellular fluid osmolality during the normalization of serum sodium concentration can cause fluid to shift into brain cells, leading to cerebral edema, which may be irreversible and potentially fatal [23]. Preterm infants may be vulnerable to hypernatremia and its neurological consequences because their capacity to regulate these processes is immature. Extremely preterm infants are particularly susceptible to these mechanisms due to their immature renal function and cerebral autoregulation, as well as greater overall clinical instability, which together may amplify the detrimental impact of hypernatremia on neurodevelopment.

This study has several limitations. First, its main limitation is the relatively small sample size (*n* = 272). The low incidence of early-onset sepsis in our cohort (*n* = 3) precluded its inclusion as a confounder. While we adjusted for potential confounders, residual confounding may have been present. Additionally, the heterogeneity within the sample, particularly the differences in gestational age, could affect the generalizability of our findings. The observed association between hypernatremia and neurodevelopmental impairment may reflect the fact that more immature infants—who are inherently more prone to hypernatremia due to their limited homeostatic capacity—are also at higher risk for poorer neurological outcomes as a result of their immaturity. Hypernatremia might serve as a marker of disease severity rather than a direct causal factor. Second, neurodevelopmental data at 3 to 4 years of age was not available for many patients, which may have introduced selection bias. This loss was more prevalent in the low-risk group, specifically among infants with higher gestational age and higher birth weight, which may have led to an overestimation of the risk ratio for neurodevelopmental outcomes in the hypernatremia group. Third, developmental impairment was assessed using the Kyoto Scale of Psychological Development rather than the widely utilized Bayley III. However, a prior study demonstrated a strong correlation between the two [15]. Fourth, hypernatremia was defined based on whole blood sodium level rather than serum sodium level, which is the standard. We acknowledge that using blood gas analyzers for sodium measurement may lead to an underestimation of serum sodium levels [28]. Despite this limitation, our findings may still be clinically valuable, as whole blood sampling requires less volume, providing a practical advantage in neonatal care. Fifth, only the presence of hypernatremia was considered, whereas its duration was not. However, to address this limitation, we performed an additional analysis in which the hypernatremia group was stratified by the number of days with hypernatremia (1 day vs. ≥ 2 days, not necessarily consecutive). Increased risks of NDI, developmental impairment, and cerebral palsy were observed only among infants with hypernatremia on ≥ 2 days (Supplementary Table 1), suggesting that prolonged hypernatremia may be associated with worse neurodevelopmental outcomes. Finally, the instrument used to measure sodium levels was changed during the study period, which may have introduced slight differences in measurement. However, a previous study reported a strong correlation between sodium levels measured by the ABL 700 and ABL 90 [29], suggesting that the impact of this change was probably minimal.

## Conclusions

Hypernatremia during the first week of life in very preterm infants was associated with neurodevelopmental impairment at 3 to 4 years of age. In particular, severe hypernatremia (> 150 mEq/L) or recurrent episodes during the first postnatal week may help identify infants at higher risk for adverse neurodevelopmental outcomes, underscoring the importance of close monitoring and early clinical intervention. Further research is warranted to determine whether preventing hypernatremia during the first week of life can improve neurodevelopmental outcomes in very preterm infants.

## Supplementary Information

Supplementary Material 1

## Data Availability

The datasets used and analyzed during the current study are available from the corresponding author on reasonable request.

## Abbreviations

CI: Confidence interval
MRI: Magnetic resonance imaging
NDI: Neurodevelopmental impairment
NE: Not estimable
NICU: Neonatal intensive care unit
RR: Risk ratio
