# Supplementary material for: Hypernatremia during the first week of life in very preterm infants and neurodevelopmental outcomes at 3 to 4 years of age: a cohort study
Source: BMC Pediatr. 2026 Feb 3;26:181. doi: 10.1186/s12887-026-06571-6 (PMC12958568; doi:10.1186/s12887-026-06571-6)
Supplement: Supplementary file 1 — Supplementary Material 1 [file 12887_2026_6571_MOESM1_ESM.docx]

| Supplementary Table 1. Associations between hypernatremia during the first week of life and neurodevelopmental outcomes, with the hypernatremia group divided into 1-day and ≥2-day hypernatremia groups, compared with the non-hypernatremia group. | | | | | | | | | |
| --- | --- | --- | --- | --- | --- | --- | --- | --- | --- |
|  |  | N case/N total | (%) |  | Crude RR | (95% CI) |  | Adjusted RR | (95% CI)^a^ |
|  | Neurodevelopmental impairment^b^ |  |  |  |  |  |  |  |  |
|  | Non-hypernatremia group | 28/190 | (14.7) |  | 1 | (reference) |  | 1 | (reference) |
|  | 1-day hypernatremia group | 6/36 | (16.7) |  | 1.13 | (0.50–2.54) |  | 1.07 | (0.50–2.28) |
|  | ≥2-day hypernatremia group | 18/46 | (39.1) |  | 2.66 | (1.61–4.37) |  | 2.37 | (1.39–4.02) |
|  | Developmental impairment^c^ |  |  |  |  |  |  |  |  |
|  | Non-hypernatremia group | 27/190 | (14.2) |  | 1 | (reference) |  | 1 | (reference) |
|  | 1-day hypernatremia group | 6/36 | (16.7) |  | 1.17 | (0.52–2.64) |  | 1.05 | (0.49–2.23) |
|  | ≥2-day hypernatremia group | 15/46 | (32.6) |  | 2.29 | (1.33–3.95) |  | 2.06 | (1.17–3.63) |
|  | Cerebral palsy |  |  |  |  |  |  |  |  |
|  | Non-hypernatremia group | 3/190 | (1.6) |  | 1 | (reference) |  | 1 | (reference) |
|  | 1-day hypernatremia group | 0/36 | (0) |  | NE |  |  | NE |  |
|  | ≥2-day hypernatremia group | 7/46 | (15.2) |  | 9.64 | (2.58–35.93) |  | 9.87 | (2.65–36.78) |
|  | Visual or hearing impairment |  |  |  |  |  |  |  |  |
|  | Non-hypernatremia group | 0/190 | (0) |  | 1 | (reference) |  | 1 | (reference) |
|  | 1-day hypernatremia group | 0/36 | (0) |  | NE |  |  | NE |  |
|  | ≥2-day hypernatremia group | 1/46 | (2.2) |  | NE |  |  | NE |  |
| Abbreviations: CI, confidence interval; NE, not estimable due to limited or no data; RR, risk ratio | | | | | | |  |  |  |
| ^a^Adjusted for gestational age, sex, small for gestational age, antenatal corticosteroids, maternal age, postnatal corticosteroids use, and non-steroidal anti-inflammatory drugs use. | | | | | | | | | |
| ^b^Infants with developmental impairment, cerebral palsy, or visual or hearing impairment. | | | | | | | | |  |
| ^c^Infants with an overall Developmental Quotient score <70 using the Kyoto Scale of Psychological Development. | | | | | | | | |  |

| Supplementary Table 2. Incidence and risk of the primary and secondary outcomes in infants with and without hypernatremia, stratified by the type of blood sodium analyzer used. | | | | | | | | | | | | | |
| --- | --- | --- | --- | --- | --- | --- | --- | --- | --- | --- | --- | --- | --- |
|  |  |  | ABL700 (2010–March 20, 2015) | | | | |  | ABL90 (March 21, 2015–2020) | | | | |
|  |  |  | Hypernatremia group | |  | Non-hypernatremia group | |  | Hypernatremia group | |  | Non-hypernatremia group | |
|  |  |  | N = 51 | |  | N = 93 | |  | N = 31 | |  | N = 97 | |
|  | Neurodevelopmental impairment^b^ | |  |  |  |  |  |  |  |  |  |  |  |
|  |  | N case/N total (%) | 18/51 | (35.3) |  | 15/93 | (16.1) |  | 6/31 | (19.4) |  | 13/97 | (13.4) |
|  |  | Adjusted RR (95% CI)^a^ | 1.66 | (0.89–3.10) |  | 1 | (reference) |  | 1.64 | (0.67–3.98) |  | 1 | (reference) |
|  | Developmental impairment^c^ | |  |  |  |  |  |  |  |  |  |  |  |
|  |  | N case/N total (%) | 15/51 | (29.4) |  | 14/93 | (15.1) |  | 6/31 | (19.4) |  | 13/97 | (13.4) |
|  |  | Adjusted RR (95% CI)^a^ | 1.49 | (0.77–2.91) |  | 1 | (reference) |  | 1.64 | (0.67–3.98) |  | 1 | (reference) |
|  | Cerebral palsy | |  |  |  |  |  |  |  |  |  |  |  |
|  |  | N case/N total (%) | 6/51 | (11.8) |  | 2/93 | (2.2) |  | 1/31 | (3.2) |  | 1/97 | (1.0) |
|  |  | Adjusted RR (95% CI)^a^ | 4.46 | (0.96–20.61) |  | 1 | (reference) |  | NE |  |  | 1 | (reference) |
|  | Visual or hearing impairment | |  |  |  |  |  |  |  |  |  |  |  |
|  |  | N case/N total (%) | 1/51 | (2.0) |  | 0/93 | (0) |  | 0/31 | (0) |  | 0/97 | (0) |
|  |  | Adjusted RR (95% CI)^a^ | NE |  |  | 1 | (reference) |  | NE |  |  | 1 | (reference) |
|  | | | | | | |  |  |  |  |  |  |  |
| Abbreviations: CI, confidence interval; NE, not estimable due to limited or no data; RR, risk ratio | | | | | | | | | | | | | |
| ^a^Adjusted for gestational age, sex, small for gestational age, antenatal corticosteroids, maternal age, postnatal corticosteroids use, and non-steroidal anti-inflammatory drugs use. | | | | | | | | | | | | | |
| ^b^Infants with developmental impairment, cerebral palsy, or visual or hearing impairment. | | | | | | | |  |  |  |  |  |  |
| ^c^Infants with an overall Developmental Quotient score <70 on the Kyoto Scale of Psychological Development. | | | | | | | | | | | | |  |
|  | | | | | | | | | | | | |  |

| Supplementary Table 3. Incidence and risk of the primary and secondary outcomes in infants with and without hypernatremia, excluding those with severe hyponatremia (<125 mEq/L, n = 14). | | | | | | | | | |
| --- | --- | --- | --- | --- | --- | --- | --- | --- | --- |
|  |  | N case/N total | (%) |  | Crude RR | (95% CI) |  | Adjusted RR | (95% CI)^a^ |
|  | Neurodevelopmental impairment^b^ |  |  |  |  |  |  |  |  |
|  | Non-hypernatremia group | 26/179 | (14.5) |  | 1 | (reference) |  | 1 | (reference) |
|  | Hypernatremia group | 22/79 | (27.8) |  | 1.92 | (1.16–3.17) |  | 1.69 | (1.00–2.84) |
|  | Developmental impairment^c^ |  |  |  |  |  |  |  |  |
|  | Non-hypernatremia group | 25/179 | (14.0) |  | 1 | (reference) |  | 1 | (reference) |
|  | Hypernatremia group | 19/79 | (24.1) |  | 1.72 | (1.01–2.94) |  | 1.48 | (0.86–2.55) |
|  | Cerebral palsy |  |  |  |  |  |  |  |  |
|  | Non-hypernatremia group | 3/179 | (1.7) |  | 1 | (reference) |  | 1 | (reference) |
|  | Hypernatremia group | 6/79 | (7.6) |  | 4.53 | (1.16–17.71) |  | 4.87 | (1.45–16.32) |
|  | Visual or hearing impairment |  |  |  |  |  |  |  |  |
|  | Non-hypernatremia group | 0/179 | (0) |  | 1 | (reference) |  | 1 | (reference) |
|  | Hypernatremia group | 1/79 | (1.3) |  | NE |  |  | NE |  |
|  | | | | | |  |  |  |  |
| CI, confidence interval; NE, not estimable due to limited or no data; RR, risk ratio | | | | | | |  |  |  |
| ^a^Adjusted for gestational age, sex, small for gestational age, antenatal corticosteroid use, maternal age, postnatal corticosteroid use, and non-steroidal anti-inflammatory drug use. | | | | | | | | | |
| ^b^Infants with developmental impairment, cerebral palsy, or visual or hearing impairment. | | | | | | |  |  |  |
| ^c^Infants with an overall Developmental Quotient score <70 on the Kyoto Scale of Psychological Development. | | | | | | | | |  |
